# Protective Efficacy of Rhesus Adenovirus COVID-19 Vaccines against Mouse-Adapted SARS-CoV-2

**DOI:** 10.1128/JVI.00974-21

**Published:** 2021-11-09

**Authors:** Lisa H. Tostanoski, Lisa E. Gralinski, David R. Martinez, Alexandra Schaefer, Shant H. Mahrokhian, Zhenfeng Li, Felix Nampanya, Huahua Wan, Jingyou Yu, Aiquan Chang, Jinyan Liu, Katherine McMahan, John D. Ventura, Kenneth H. Dinnon, Sarah R. Leist, Ralph S. Baric, Dan H. Barouch

**Affiliations:** a Center for Virology and Vaccine Research, Beth Israel Deaconess Medical Center, Harvard Medical School, Boston, Massachusetts, USA; b Department of Epidemiology, University of North Carolina at Chapel Hill, Chapel Hill, North Carolina, USA; c Ragon Institute of MGH, MIT and Harvard, Cambridge, Massachusetts, USA; d Harvard Medical School, Boston, Massachusetts, USA; e Massachusetts Consortium on Pathogen Readiness, Boston, Massachusetts, USA; Loyola University Chicago

**Keywords:** COVID-19, SARS-CoV-2, adenoviruses, live vector vaccines

## Abstract

The global COVID-19 pandemic has sparked intense interest in the rapid development of vaccines as well as animal models to evaluate vaccine candidates and to define immune correlates of protection. We recently reported a mouse-adapted SARS-CoV-2 virus strain (MA10) with the potential to infect wild-type laboratory mice, driving high levels of viral replication in respiratory tract tissues as well as severe clinical and respiratory symptoms, aspects of COVID-19 disease in humans that are important to capture in model systems. We evaluated the immunogenicity and protective efficacy of novel rhesus adenovirus serotype 52 (RhAd52) vaccines against MA10 challenge in mice. Baseline seroprevalence is lower for rhesus adenovirus vectors than for human or chimpanzee adenovirus vectors, making these vectors attractive candidates for vaccine development. We observed that RhAd52 vaccines elicited robust binding and neutralizing antibody titers, which inversely correlated with viral replication after challenge. These data support the development of RhAd52 vaccines and the use of the MA10 challenge virus to screen novel vaccine candidates and to study the immunologic mechanisms that underscore protection from SARS-CoV-2 challenge in wild-type mice.

**IMPORTANCE** We have developed a series of SARS-CoV-2 vaccines using rhesus adenovirus serotype 52 (RhAd52) vectors, which exhibit a lower seroprevalence than human and chimpanzee vectors, supporting their development as novel vaccine vectors or as an alternative adenovirus (Ad) vector for boosting. We sought to test these vaccines using a recently reported mouse-adapted SARS-CoV-2 (MA10) virus to (i) evaluate the protective efficacy of RhAd52 vaccines and (ii) further characterize this mouse-adapted challenge model and probe immune correlates of protection. We demonstrate that RhAd52 vaccines elicit robust SARS-CoV-2-specific antibody responses and protect against clinical disease and viral replication in the lungs. Further, binding and neutralizing antibody titers correlated with protective efficacy. These data validate the MA10 mouse model as a useful tool to screen and study novel vaccine candidates, as well as the development of RhAd52 vaccines for COVID-19.

## INTRODUCTION

A critical component of the evaluation of vaccine candidates for COVID-19 has been the development of preclinical challenge models. Transgenic mice (1–5), hamsters (6–9), and nonhuman primates (10–12) have been shown to support viral replication and, to various degrees, clinical disease following infection with SARS-CoV-2 (13). We recently described a mouse-adapted virus (MA10) to enable challenge of standard, wild-type laboratory mice and recapitulate several key features of human disease, such as viral replication in respiratory tract tissues and severe infection-associated weight loss (14). This model has been explored to evaluate small molecule antivirals and candidate monoclonal antibodies for prophylactic or therapeutic applications, as well as prototype vaccine candidates (14–17). For example, initial studies using viral replicon particles expressing SARS-CoV-2 spike protein demonstrate the capacity of vaccines to restrain MA10 infection and disease (14).

This model has not yet been utilized to study the characteristics of vaccine-elicited immune responses that protect against clinical disease and viral replication. Thus, we sought to test a series of candidate rhesus adenovirus serotype 52 (RhAd52) (18) vector-based vaccines expressing engineered versions of SARS-CoV-2 spike. RhAd52 vectors have lower seroprevalence in human populations than Ad26 vectors, which recently received FDA emergency use authorization as a COVID-19 vaccine (19, 20). Further, we hypothesized that testing this series of vaccine inserts could generate a range of immune responses, with various magnitudes of humoral immune responses. We sought to harness this approach to probe correlates of protection, including whether similar immune parameters such as neutralizing antibody titers emerge in the mouse model as predictors of challenge outcome, as has been observed in hamsters and nonhuman primates (9, 10, 21). These data will inform applications of the MA10 virus to study key questions about clinical disease, infection, or both.

## RESULTS

### Immunogenicity of RhAd52 vectors.

**FIG 1:**
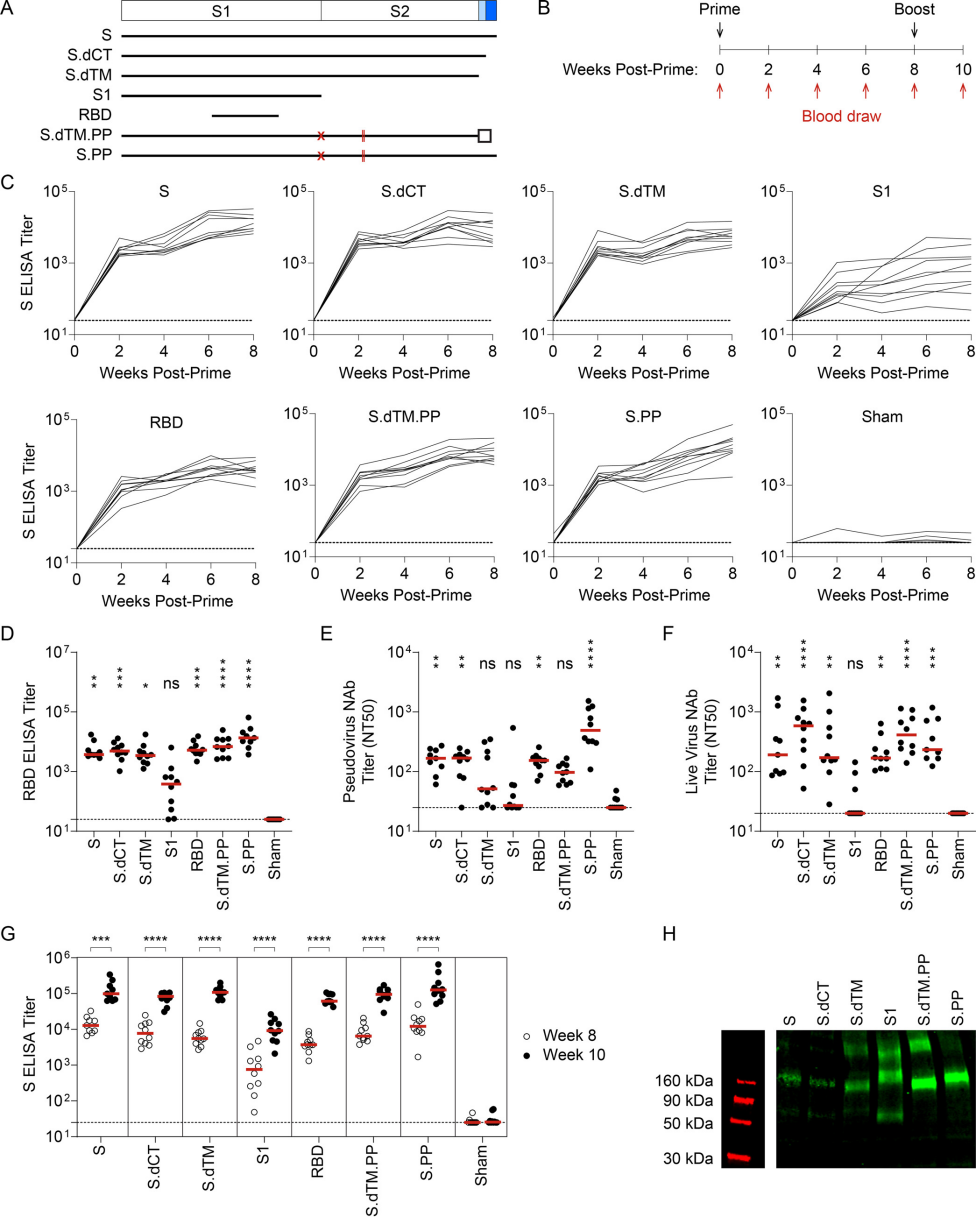
RhAd52 vaccines elicit robust S-specific binding antibody responses. (A) A series of replication-incompetent RhAd52 vectors, encoding variations on the SARS-CoV-2 spike (S) protein, was designed. Inserts included (i) S, (ii) deletion of the cytoplasmic tail (S.dCT), (iii) deletion of the transmembrane domain and cytoplasmic tail (S.dTM), (iv) the S1 domain with a foldon trimerization tag, (v) the receptor binding domain (RBD) with a foldon trimerization tag, (vi) deletion of the transmembrane domain and cytoplasmic tail, with mutation of the furin cleavage site (red X), addition of stabilizing proline mutations (red lines), and a foldon trimerization tag (S.dTM.PP), and (vii) S with mutation of the furin cleavage site (red X) and addition of stabilizing proline mutations (red lines) (S.PP). (B) To explore the immunogenicity of these vaccine candidates, wild-type BALB/c mice were immunized at week 0 with 10^9^ viral particles (VPs) of candidate RhAd52 vaccines or sham. Peripheral blood was collected at baseline and every 2 weeks following vaccination to monitor antibody responses in serum. Eight weeks postprime, mice were administered a homologous boost to explore the potential to boost responses. (C) For each RhAd52 insert, as well as sham controls, S-specific binding antibody responses were quantified through enzyme-linked immunosorbent assay (ELISA) in serum every 2 weeks postprime. (D) The distribution of RBD-specific ELISA titers at week 8 across candidate RhAd52 vectors. Red lines indicate the median titer of each group. (E and F) Neutralizing activity of vaccine-elicited antibody responses were assessed through (E) pseudovirus or (F) live SARS-CoV-2 virus *in vitro* neutralization assays. The 50% neutralization titer (NT50) is displayed, with the median of each vaccine regimen indicated with a red line. In panels D to F, statistics displayed are the results of a Kruskal-Wallis test with Dunn’s test for multiple comparisons, comparing experimental groups to the sham control (ns, no significant difference; *, *P* ≤ 0.05; **, *P* ≤ 0.01; ***, *P* ≤ 0.001; ****, *P* ≤ 0.0001). (G) The distribution of S-specific ELISA titers at week 8 (open circles) and week 10 (2 weeks postboost, closed circles) was measured to characterize the immunogenicity of a homologous boost with candidate RhAd52 vaccines. In panel G, to focus the analyses on the effect of the boost for each vaccine insert, the statistics shown are the results of two-sided Mann-Whitney tests comparing pre- and postboost titers for each candidate vaccine. In panels C to G, red lines indicate the median titer of each group. *n* = 9 to 10 mice/group, and endpoint binding titers are reported. Representative data from one of two similar experiments are shown. (H) Western blot analyses of the *in vitro* expression of the indicated vaccine insert in HEK293T cells.

We designed a series of replication-incompetent viral vector vaccines using rhesus adenovirus serotype 52 (RhAd52) vectors (18, 22) that encode variations of the SARS-CoV-2 spike (S) protein (Fig. 1A). Similar to our previous reports with human adenovirus serotype 26 (Ad26) vectors (9, 23, 24), inserts included: (i) unmodified S, (ii) truncations of the cytoplasmic tail (S.dCT) or the transmembrane region (S.dTM), or (iii) select fragments, including the S1 domain and the receptor binding domain (RBD). In some cases, immunogens were modified with mutation of the furin cleavage site and the addition of proline mutations to stabilize protein prefusion conformation (PP) (25–27). To explore the potential of candidate RhAd52 vaccines to elicit humoral immune responses, groups of wild-type BALB/c mice were immunized with 10^9^ viral particles (VPs) of these vaccines via the intramuscular route (Fig. 1B). Peripheral blood was collected on a biweekly basis to monitor antibody responses in serum.

At week 2 following the initial immunization, 100% of mice immunized with RhAd52 vaccines, irrespective of the immunogen insert, exhibited SARS-CoV-2 S-specific binding antibodies by enzyme-linked immunosorbent assay (ELISA) (Fig. 1C). These responses generally increased over the time frame of 2 to 8 weeks post-prime. At week 8, RBD-specific binding responses were also observed in 100% of mice immunized with RhAd52 candidates (Fig. 1D). Furthermore, antibody function was assessed using *in vitro* assays to quantify the potential to neutralize either a pseudotyped virus (10, 28, 29) (Fig. 1E) or live SARS-CoV-2 virus (10, 28, 30, 31) (Fig. 1F). Similar to the binding results, neutralizing titers were elicited by several of the RhAd52 candidate vaccines, with the lowest responses observed following immunization with the S1 domain insert, in which a subset of mice exhibited no detectable neutralizing responses at week 8. *In vitro* assays suggest that observed attenuated immune responses to S1 are not solely a result of lower expression of the S1 construct relative to other inserts (Fig. 1H). However, future studies could explore these questions in more detail. Mice received a second identical dose of the respective RhAd52 vectors at week 8. Two weeks following the boost immunization, median S-specific ELISA titers were found to increase by approximately 10-fold for all the vaccine candidates (Fig. 1G). These data demonstrate the immunogenicity of a homologous boost with a second immunization of a RhAd52 vector.

**FIG 2:**
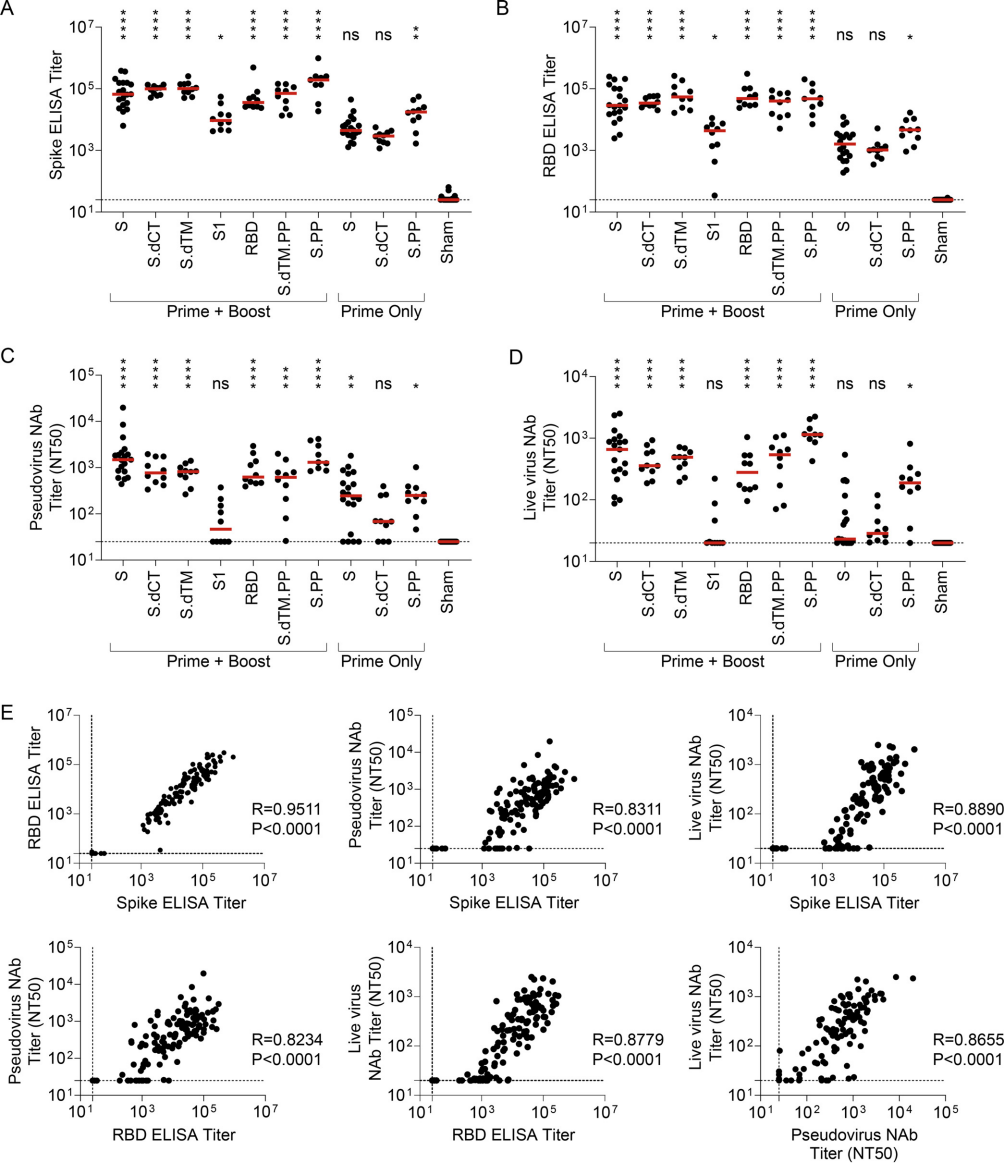
Serum binding and neutralizing antibody responses are tightly linked following RhAd52 vaccination. Groups of mice were administered a prime (week 0) and a boost (week 8) of 10^9^ VP the indicated RhAd52 vaccines. At the time of boost (i.e., week 8), additional groups of mice were administered a single dose (i.e., prime only) of 10^9^ VP of select indicated RhAd52 vaccines. At week 12 (i.e., 4 weeks postboost or 4 weeks post-prime only), serum was analyzed. (A) S-specific and (B) RBD-specific ELISA titers are shown, with the median titer for each regimen indicated with a red line. (C and D) Neutralizing activity of vaccine-elicited antibody responses was assessed through (C) pseudovirus or (D) live SARS-CoV-2 virus neutralization assays. The 50% neutralization titer (NT50) is displayed, with the median of each vaccine regimen indicated with a red line. In panels A to D, the statistics displayed are the results of a Kruskal-Wallis test with Dunn’s test for multiple comparisons, comparing experimental groups to the sham control (ns, no significant difference; *, *P* ≤ 0.05; **, *P* ≤ 0.01; ***, *P* ≤ 0.001; ****, *P* ≤ 0.0001). (E) Spearman correlation analyses of binding and neutralizing antibody responses are displayed. For panels A to E, data are pooled from two similar experiments. For panels A to D, *n* = 9 to 10 mice/group for all regimens, with the exception of RhAd52.S prime + boost (*n* = 19), RhAd52.S prime only (*n* = 20), and sham (*n* = 23). In panel E, data from all vaccine regimens are pooled to explore the relationship between binding and neutralizing antibody function independent of the RhAd52 insert.

We next designed a series of immunization regimens that we hypothesized would (i) allow direct comparison of protective efficacy of single-shot versus two-dose prime-boost schedules and (ii) generate a range of binding and neutralizing antibody responses that could enable analyses of correlates of protection following challenge (21, 24, 28). Briefly, groups of mice were immunized with a prime and a matched boost with the seven candidate RhAd52 vaccines, as in Fig. 1B. At the time of boost (i.e., week 8), additional groups of mice were immunized with a single dose of select vaccines, RhAd52.S, RhAd52.S.dCT, and RhAd52.S.PP. At week 12, serum was collected to assess antibody responses prior to viral challenge. Expansion of S-specific (Fig. 2A) and RBD-specific (Fig. 2B) binding antibody titers was again observed in all vaccinated mice. Groups of mice that received the two-dose regimens exhibited approximately 1-log-higher median titers than groups administered a single immunization. Furthermore, consistent with our previous data using a DNA vaccination platform in nonhuman primates (28), the S1 insert drove the lowest binding responses.

In evaluating neutralizing antibody activity elicited by these vaccine regimens, similar patterns were observed with pseudovirus (Fig. 2C) and live virus (Fig. 2D) assays. Among the groups administered prime-boost dosing schedules, high neutralizing antibody titers were observed across all vaccines with the exception of the S1 immunogen, for which 50% neutralization titer (NT50) values were below the assay limit of detection for several mice. Groups that received only one dose of select inserts exhibited lower neutralizing titers than boosted mice, with detectable responses in a subset of mice that, on average, were approximately 1-log lower than the boosted groups that received full-length or truncated (e.g., S.dCT) vaccines. As consistent trends were observed across binding and neutralizing titers in the relative magnitude of responses elicited by various candidate vaccines, correlation analyses were performed to assess the relationship between these immunologic readouts (Fig. 2E). Highly significant (*P* < 0.0001, Spearman correlation) strong positive correlations were observed between binding ELISA titers to S and RBD proteins and capacity to neutralize either pseudovirus or live SARS-CoV-2 virus.

### Protective efficacy of RhAd52 vector vaccines against MA10 challenge.

**FIG 3:**
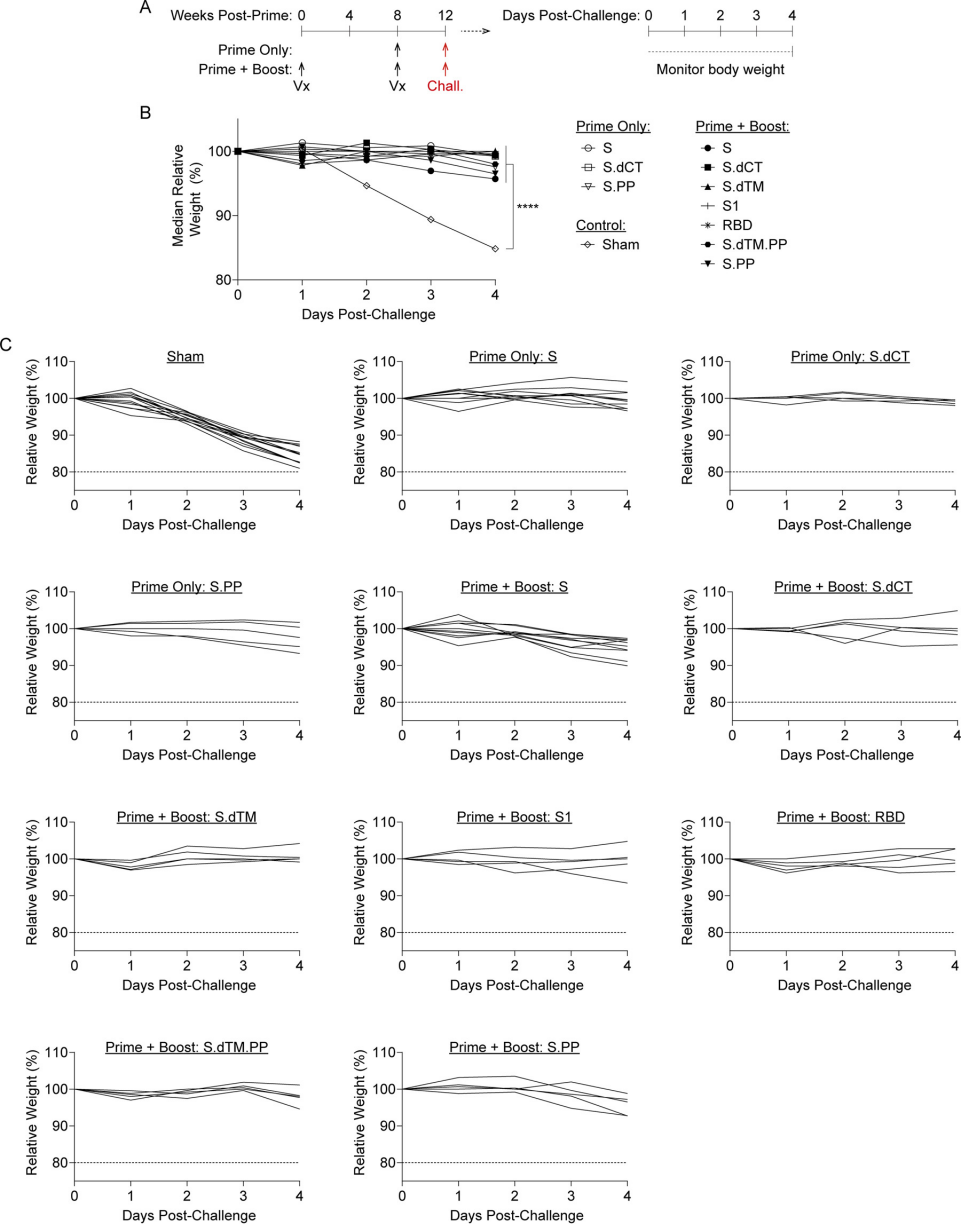
RhAd52 vaccines protect from clinical disease following mouse-adapted SARS-CoV-2 challenge. (A) Groups of mice were immunized with either a prime and boost or a prime only of 10^9^ viral particles (VPs) of RhAd52 candidate vaccines following the indicated timeline. At week 12, mice were challenged with 10^4^ PFU of MA10 SARS-CoV-2 via the intranasal route. After challenge, a subset of mice was followed through day 4 postchallenge to monitor for signs of clinical disease. (B) Relative body weight following MA10 SARS-CoV-2 challenge in mice immunized with the indicated vaccine regimens. The median value of each group is displayed. ****, *P* < 0.0001 (results of a one-way ANOVA followed by Dunnett’s multiple-comparison test, comparing vaccinated groups to the sham control group). (C) Traces of relative body weight in individual mice, immunized with the indicated RhAd52 vaccine regimen, following challenge. For panels B and C, data are pooled from two similar experiments. *n* = 5 mice/group for all regimens, with the exception of RhAd52.S prime + boost (*n* = 10), RhAd52.S prime only (*n* = 10), and sham (*n* = 13).

At week 12, all groups of mice were challenged to evaluate whether vaccine-elicited responses protected from clinical disease and viral replication in this mouse-adapted model (14). Vaccinated mice were challenged on day 0 with 10^4^ PFU SARS-CoV-2 MA10 via the intranasal route (Fig. 3A). Half of the mice were followed through day 4 postchallenge, and body weight was monitored daily for signs of clinical disease. At the terminal time point, lung tissue was collected, and outgrowth assays were performed to quantify replication-competent virus (i.e., PFU) in this key respiratory tract tissue. In parallel, half of the mice were sacrificed at day 2 postchallenge to measure virus in the lungs. We hypothesized this approach would allow evaluation of the potential to restrain clinical symptoms of disease as well as enable a virologic endpoint at the time of typical peak viral load (i.e., day 2 postchallenge).

**FIG 4:**
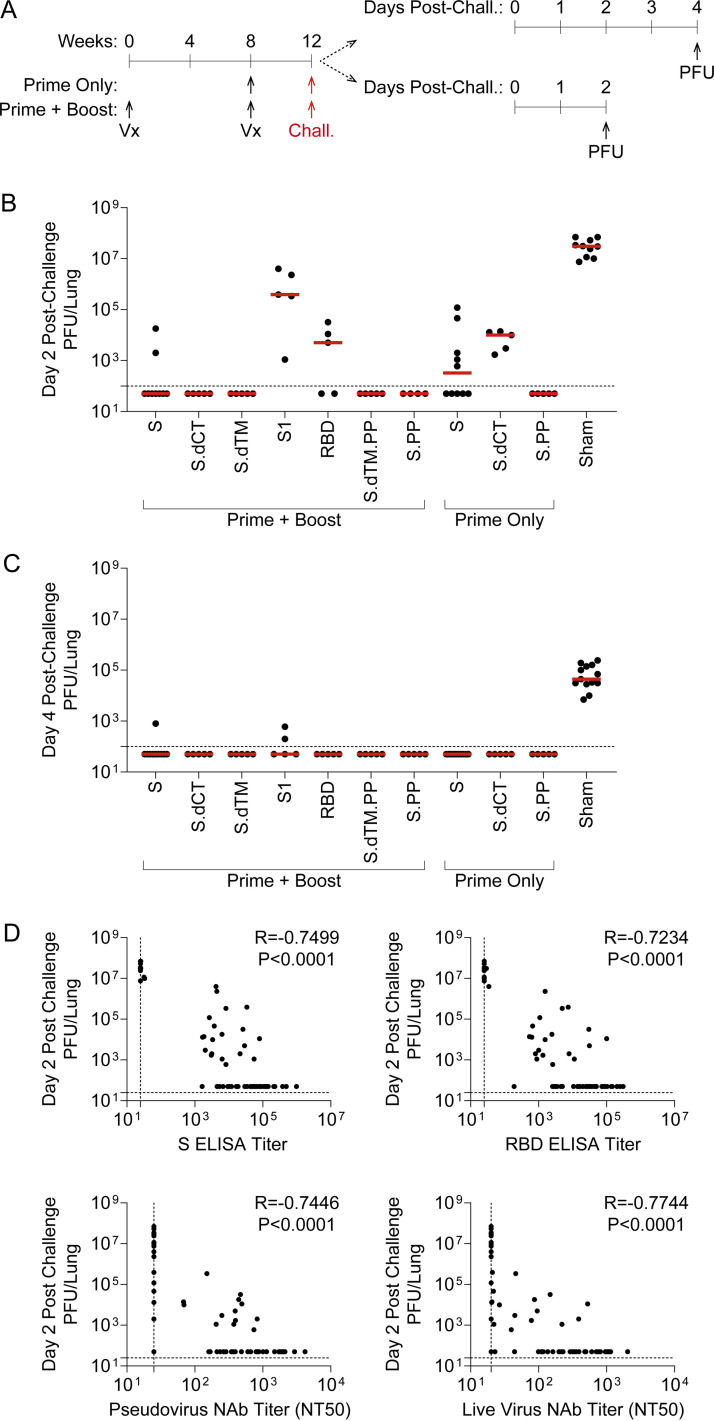
RhAd52 vaccine-elicited antibody responses link to restraint of viral replication in the lung following mouse-adapted SARS-CoV-2 challenge. (A) Groups of mice were immunized with either a prime and a boost or a prime only of 10^9^ viral particles (VPs) of RhAd52 candidate vaccines. At week 12, mice were challenged with 10^4^ PFU of MA10 SARS-CoV-2 via the intranasal route. A subset of mice was monitored through day 4 postchallenge; at the terminal time point, lungs were harvested to measure virus via outgrowth assays to quantify PFU per tissue. The second subset of mice were followed through day 2 postchallenge for similar PFU assays at the time of peak viral replication. (B and C) Quantification of PFU per lung at (B) day 2 and (C) day 4 postchallenge. The median of each group is indicated by the red line. *n* = 4 to 5 mice/group for all regimens, with the exception of RhAd52.S prime + boost (*n* = 9), RhAd52.S prime only (*n* = 10), and sham (*n* = 10). (D) Spearman correlation analyses of prechallenge serum binding or neutralizing antibody responses with day 2 postchallenge viral titers in lung are displayed. For panels B to C, data are pooled from two similar experiments. In panel E, data from all vaccine regimens are pooled to explore the relationship between prechallenge binding and neutralizing antibody responses and the virologic endpoint independent of the RhAd52 insert.

As expected, the sham control group exhibited significant weight loss following MA10 challenge, with a median loss of 15.2% of body weight at day 4 postchallenge (Fig. 3B and C). All vaccine regimens provided robust protection from clinical signs of infection in terms of body weights (*P* < 0.0001, one-way analysis of variance [ANOVA] with Dunnett’s multiple-comparison test), with body weight generally remaining stable irrespective of the RhAd52 insert or whether a single or two-dose vaccine regimen was employed. Analyses of lungs revealed differences in the level of replication-competent virus detected in respiratory tract tissues among mice largely protected from weight loss (Fig. 4A). In sham control mice at day 2 postchallenge, high levels of virus were recovered from lung, with a median titer of 3.1 × 10^7^ PFU/lung (Fig. 4B). In contrast, two-dose regimens with full-length (i.e., S, S.PP) or truncated (i.e., S.dCT, S.dTM, S.dTM.PP) S immunogens provided a dramatic reduction in viral titer, with a greater than a 6-log drop in median titer. In nearly all mice in these groups, no replication-competent virus was recovered from the lungs (i.e., PFU, <100/lung). Immunization with two doses of the S fragment immunogens—RhAd52.S1 and RhAd52.RBD—restrained the level of virus in the lung, with a median titer of 3.9 × 10^5^ and 5.0 × 10^3^ PFU/lung, respectively. Similarly, in the single-shot groups, RhAd52.S and RhAd52.S.dCT provided significant but incomplete protection, reducing the viral burden in the lung to 3.3 × 10^2^ and 1.0 × 10^4^ PFU/lung, respectively. Finally, a single shot of RhAd52.S.PP dramatically reduced viral load, with no detectable viral outgrowth from lung tissues in 100% of mice. Of note, the S.PP insert previously proved optimal in nonhuman primates (24) and was advanced into clinical trials (19, 20).

Similar analyses at day 4 revealed the level of virus in lung tissue was several logs lower (median, 4.4 × 10^4^ PFU/lung) than at day 2 postchallenge (Fig. 4C), consistent with our previous finding that tissue viral loads peak at day 1 to 2 postchallenge and gradually resolve over approximately 7 days (14). Across vaccine regimens, virus levels were largely below the limit of detection of the outgrowth assay, with low levels observed in a subset of mice in the RhAd52.S and RhAd52.S1 two-dose groups. Together, these data suggest that all vaccines led to a reduction in respiratory tract tissue viral loads at the typical peak of infection as well as significantly decreased the persistence of virus in the lungs.

### Exploration of immune correlates of protection.

We next evaluated possible correlations between vaccine-elicited immune responses prior to challenge and peak viral levels following challenge. A highly significant (*P* < 0.0001) correlation was observed between prechallenge S ELISA titers and peak (i.e., day 2) lung PFU (Fig. 4D). Furthermore, the resulting Spearman correlation coefficient (*R* = –0.7499) suggests a strong inverse relationship between prechallenge binding antibody levels and virologic outcome postchallenge. Similar highly significant (*P* < 0.0001) inverse correlations were observed between three additional prechallenge immunologic metrics and viral replication in the lung: (i) RBD ELISA titers (*R* = –0.7234), (ii) pseudovirus neutralization titers (*R* = –0.7446, and (iii) live virus neutralization titers (*R* = –0.7744). We did observe that the pseudovirus and live virus neutralization assays appeared to have lower sensitivity than the binding assays, with a fraction of vaccinated animals exhibiting detectable binding, but not detectable neutralizing titers. This observation may limit the correlations of neutralizing assays in the context of low-level immune responses. Taken together, these data suggest that multiple vaccine-elicited humoral immune responses are inversely correlated with viral replication in respiratory tract tissue following MA10 SARS-CoV-2 challenge.

## DISCUSSION

Our data indicate that RhAd52 vectors expressing SARS-CoV-2 S antigens elicit robust and protective humoral immune responses in mice. Based on baseline seroprevalence as well as the expanded global use of Ad5, Ad26, and ChAdOx1 vaccines (19, 20, 32–35), developing additional adenoviral vectors for COVID-19 vaccines is critical. This approach could be important for developing future boosting vectors or to tune the innate immune signatures that are induced (36). Similar to our recent reports in hamsters (9), nonhuman primates (24, 28), and humans (19), a robust correlation was observed between binding and neutralizing antibody responses. Furthermore, although single-shot vaccines were highly protective, we observed increased immune responses using a homologous prime-boost strategy. In particular, the expansion of neutralizing antibody responses, as measured by both pseudovirus and live virus assays, in mice is encouraging, as this metric has emerged as a potential correlate of protection in hamster and nonhuman primate challenge models (9, 10, 21, 24, 28).

Importantly, the MA10 virus has previously been shown to drive significant clinical disease (i.e., weight loss) as well as replication localized in respiratory tract tissues, characteristics of interest for modeling severe COVID-19 disease. In contrast, nonhuman primate models for COVID-19 generally do not develop severe clinical disease (10–13). The MA10 mouse model has proven useful for screening candidate therapeutics, but it remains relatively unexplored for testing vaccines (14–17). The results from our challenge studies using RhAd52 vaccines suggest that candidate vaccines significantly protected against clinical disease and virus replication in lung tissue. However, only select immunization regimens drove full suppression of replicating virus in the lungs, as measured by viral outgrowth assays. Moreover, our data show that the recently reported mouse-adapted virus MA10 exhibits robust humoral immune correlates of vaccine protection (9, 10, 21), which will prove useful in future studies of vaccines and other interventions using this model.

Future studies could further explore mechanistic correlates of protection, such as defining how vaccine candidates tune systemic proinflammatory cytokine secretion typically triggered by viral infection or the potential to prevent disease pathology in the lung and upper respiratory tract, which were not explored in our current experiments. Further, our studies largely centered on binding and neutralizing antibody responses as immune correlates of protection; future studies could explore the role of T cell responses in the context of MA10 challenge. No signs of disease enhancement (e.g., enhanced weight loss) were observed with subprotective immune responses, an important finding due to concerns of antibody-dependent enhancement. Together, these data support the MA10 mouse-adapted virus as a tool to screen vaccine candidates. This approach could help to test novel immunogens, delivery systems, or dosing regimens, harnessing a relatively high-throughput, tractable small animal model, wild-type mice. These studies could be employed to identify promising approaches to advance to alternative preclinical models, including hamsters and nonhuman primates and, subsequently, early clinical trials. Moreover, similar mouse challenge models could be developed for the newly described SARS-CoV-2 variants of concern.

## MATERIALS AND METHODS

### RhAd52 vectors.

RhAd52 vectors were constructed with seven variants of the SARS-CoV-2 spike (S) protein sequence (Wuhan/WIV04/2019; GenBank accession number MN996528.1). Sequences were codon optimized and synthesized. Replication-incompetent, E1/E3-deleted RhAd52-vectors were produced in HEK293B-55K.TetR cells as previously described (23), with the E1 region replaced by a transgene cassette encoding the S sequence of interest. Vectors were sequenced and tested for expression before use.

### Western blot.

For Western blot analysis, HEK293T cells (4.8 × 10^5^ cells/well in six-well plates) were transduced with 1 × 10^11^ viral particles of Ad26 vectors encoding SARS-CoV-2 spike transgenes. Cell lysates were prepared by adding radioimmunoprecipitation assay (RIPA) buffer (Thermo Fisher) to cells for 10 min 72 h after transduction. Cell lysate samples were mixed with 4X protein sample loading buffer (Li-COR). After heating for 5 min at 90°C, samples were loaded on a precast 4 to 20% SDS-PAGE gel (Bio-Rad). Proteins were transferred to a polyvinylidene difluoride (PVDF) membrane using an iBlot dry blotting system (Invitrogen), and membrane blocking was performed for 1 h at room temperature in intercept blocking buffer (Li-COR). After blocking, the membrane was incubated overnight at 4°C with a 1:5,000 dilution of polyclonal rabbit anti-SARS-CoV-2 RBD antibody (Sino Biological) in intercept blocking buffer plus 0.2% Tween. After incubation, the membrane was washed four times with phosphate-buffered saline (PBS) plus 0.2% Tween for 5 min and subsequently incubated for 1 h with 1:10,000 IRDye 800CW-conjugated goat-anti-rabbit secondary antibody (Li-COR) in Tris-buffered saline with Tween 20 (TBST)-5% blocker. Finally, the PVDF membrane was washed four times with PBS plus 0.2% Tween plus 0.01% SDS for 5 min, washed once with PBS, and imaged using an Odyssey CLx infrared imaging system (Li-COR).

### Animals and study design.

Female BALB/c mice (The Jackson Laboratory) were randomly allocated to groups. Mice received RhAd52 vectors expressing different versions of the SARS-CoV-2 S protein or sham controls (*n *= 10 per group). Animals received a single immunization of 10^9^ viral particles (VPs) of RhAd52 vectors by the intramuscular route without adjuvant. In some cases, 8 weeks later, mice received a homologous boost immunization. At the indicated time points, peripheral blood was collected via the submandibular route to isolate serum for immunologic assays. For viral challenge, mice were administered 1 × 10^4^ PFU MA10 SARS-CoV-2 in a volume of 50 μl via the intranasal route (14). Following challenge, body weights were assessed daily. Subsets of animals were euthanized on days 2 and 4 postchallenge for viral outgrowth assays. All animal studies were conducted in compliance with all relevant local, state, and federal regulations and were approved by the Beth Israel Deaconess Medical Center and University of North Carolina at Chapel Hill Institutional Animal Care and Use Committees.

### ELISA.

S- and RBD-specific binding antibodies were assessed by ELISA essentially as described (10, 28). Briefly, plates were coated with 1 μg ml^−1^ of SARS-CoV-2 S protein (Sino Biological) or SARS-CoV-2 RBD protein (Aaron Schmidt, Massachusetts Consortium on Pathogen Readiness), diluted in 1× PBS, and incubated at 4°C overnight. After incubation, plates were washed once with a wash buffer (0.05% Tween 20 in 1× PBS) and blocked with 350 μl of casein per well. The block solution was discarded after 2 to 3 h of incubation at room temperature, and plates were blotted dry. Then, 3-fold serial dilutions of mouse serum in casein block were added to the wells, and the plates were incubated for 1 h at room temperature. The plates were then washed three times, and rabbit anti-mouse IgG HRP (Jackson ImmunoResearch), diluted 1:1,000 in casein block, was added to the wells and incubated at room temperature in the dark. After 1 h, the plates were washed three times, and 100 μl of SeraCare Kirkegaard & Perry Laboratories (KPL) TMB (3,3′,5,5′-tetramethylbenzidine) SureBlue start solution was added to each well. Development was halted with the addition of 100 μl of SeraCare KPL TMB stop solution per well. The absorbance at 450 nm was recorded using a VersaMax microplate reader. ELISA endpoint titers were defined as the highest reciprocal serum dilution that yielded an absorbance of >0.2. The raw optical density (OD) values were transferred into GraphPad Prism for analysis. A standard curve was interpolated using a sigmoidal four-parameter logistic (4PL) fit. To quantify the endpoint titer, the interpolation function was used to calculate the dilution at which the OD value would be equal to a value of 0.2.

### Pseudovirus neutralization assay.

A SARS-CoV-2 pseudovirus expressing a luciferase reporter gene was generated in an approach similar to that described previously (10, 28, 29). Briefly, the packaging construct psPAX2 (AIDS Resource and Reagent Program), luciferase reporter plasmid pLenti-CMV Puro-Luc (Addgene), and S protein expressing pcDNA3.1-SARS CoV-2 S.dCT were cotransfected into HEK293T cells using lipofectamine 2000 (Thermo Fisher Scientific). After 48 h, supernatant was collected, and pseudotyped viruses were purified by filtration with a 0.45-μm filter. To determine the neutralization activity of the antisera from vaccinated animals, HEK293T-hACE2 target cells were seeded in 96-well tissue culture plates at a density of 1.75 × 10^4^ cells per well and incubated overnight. Then, 3-fold serial dilutions of heat-inactivated serum were prepared and mixed with 50 μl of pseudovirus. The mixture was incubated at 37°C for 1 h before being added to HEK293T-hACE2 cells. Then, 48 h after infection, cells were lysed in Steady-Glo luciferase (Promega) according to the manufacturer’s instructions. Neutralization titers were defined as the sample dilution at which a 50% reduction in relative light units was observed relative to the average of the virus control wells.

### Live virus neutralization assay.

Live virus neutralization of sera was determined using a nanoLuciferase-expressing SARS-CoV-2 virus (SARS-CoV-2nLuc), bearing wild-type spike protein, as described (37, 38), with slight modification. Briefly, Vero E6 cells were seeded at 2 × 10^4^ cells per well in a 96-well plate 24 h before the assay. Next, 90 PFU of SARS-CoV-2-nLuc virus were mixed with serial-diluted sera at 1:1 ratio and incubated at 37°C for 1 h. An 8-point, 3-fold dilution curve was generated for each sample, with starting a concentration of 1:20. The virus and serum mix was added to cells and incubated at 37°C + 5% CO_2_ for 48 h. Luciferase activity was measured with a Nano-Glo luciferase assay system (Promega) following the manufacturer’s protocol using a SpectraMax M3 luminometer (Molecular Devices). Then, 50% neutralization titer (NT50) was calculated with GraphPad Prism by fitting the data points to a sigmoidal dose-response (variable slope) curve.

### PFU assay.

Lung viral titers were determined by plaque assay. Briefly, right caudal lung lobes were homogenized in 1 ml PBS using glass beads, and serial dilutions of the clarified lung homogenates were added to a monolayer of Vero E6 cells and overlaid with a solution of 0.8% agarose and medium. After 3 days, plaques were visualized via staining with neutral red dye and counted.
